# SNPs rs11240569, rs708727, and rs823156 in *SLC41A1* Do Not Discriminate Between Slovak Patients with Idiopathic Parkinson’s Disease and Healthy Controls: Statistics and Machine-Learning Evidence

**DOI:** 10.3390/ijms20194688

**Published:** 2019-09-21

**Authors:** Michal Cibulka, Maria Brodnanova, Marian Grendar, Milan Grofik, Egon Kurca, Ivana Pilchova, Oto Osina, Zuzana Tatarkova, Dusan Dobrota, Martin Kolisek

**Affiliations:** 1 Division of Neurosciences, Biomedical Center Martin, Jessenius Faculty of Medicine in Martin, Comenius University in Bratislava, 03601 Martin, Slovakia; 2 Department of Medical Biochemistry, Jessenius Faculty of Medicine in Martin, Comenius University in Bratislava, 03601 Martin, Slovakia; 3 Department of Bioinformatics, Biomedical Center Martin, Jessenius Faculty of Medicine in Martin, Comenius University in Bratislava, 03601 Martin, Slovakia; 4 Clinic of Neurology, University Hospital in Martin, 03601 Martin, Slovakia; 5 Clinic of Occupational Medicine and Toxicology, University Hospital in Martin, 03601 Martin, Slovakia

**Keywords:** Parkinson’s disease, locus *PARK16*, SLC41A1, Na^+^/Mg^2+^ exchanger, polymorphism, machine-learning

## Abstract

Gene *SLC41A1* (*A1*) is localized within Parkinson’s disease-(PD)-susceptibility locus *PARK16* and encodes for the Na^+^/Mg^2+^-exchanger. The association of several *A1* SNPs with PD has been studied. Two, rs11240569 and rs823156, have been associated with reduced PD-susceptibility primarily in Asian populations. Here, we examined the association of rs11240569, rs708727, and rs823156 with PD in the Slovak population and their power to discriminate between PD patients and healthy controls. The study included 150 PD patients and 120 controls. Genotyping was performed with the TaqMan^®^ approach. Data were analyzed by conventional statistics and Random Forest machine-learning (ML) algorithm. Individually, none of the three SNPs is associated with an altered risk for PD-onset in Slovaks. However, a combination of genotypes of SNP-triplet GG_(rs11240569)_/AG_(rs708727)_/AA_(rs823156)_ is significantly (*p* < 0.05) more frequent in the PD (13.3%) than in the control (5%) cohort. ML identified the power of the tested SNPs in isolation or of their singlets (joined), duplets and triplets to discriminate between PD-patients and healthy controls as zero. Our data further substantiate differences between diverse populations regarding the association of *A1* polymorphisms with PD-susceptibility. Lack of power of the tested SNPs to discriminate between PD and healthy cases render their clinical/diagnostic relevance in the Slovak population negligible.

## 1. Introduction

Parkinson’s disease (PD) is the second most prevalent, slowly progressing neurodegenerative disorder and begins years ahead of the onset of diagnosable symptoms [1,2,3]. It involves multiple neuroanatomical areas, results from a combination of genetic and environmental factors, and manifests with a broad range of symptoms, as summarized in Table A1 [3].

Magnesium (Mg) plays an essential role in a plethora of biochemical and physiological processes in cells and organisms [4,5]. Deficiency of Mg may, in the long term, affect the central nervous system [6]. Moreover, intracellular Mg deficiency in brain cells is thought to contribute to the onset of neurodegenerative disorders such as PD in man [7,8,9].

**Figure 1:**
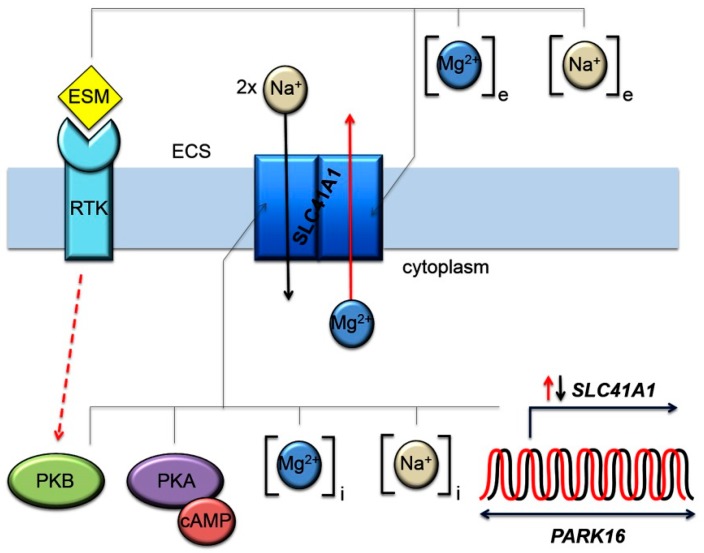
Factors regulating activity of Na^+^/Mg^2+^ exchange via SLC41A1 (A1). Among the most prominent intracellular A1 activity-regulating factors belong: cAMP-dependent PKA (activator), Akt/PKB (inhibitory effect via activation of cAMP degradation by phosphodiesterase 3b (PDE3b)) [14], transcription activity of *A1*, and intracellular concentrations of Na^+^ and Mg^2+^. The regulation by extracellular factors such as concentrations of Na^+^ and Mg^2+^ in the extracellular fluid and extracellular signaling molecules/hormones (ESM; e.g., insulin, neuritin, PDGF, EGF), which stimulate various receptor tyrosine kinases (RTK) are also equally important. RTK further activate adjacent signaling cascades (represented by dashed red arrow) that merge together in PI3K–Akt/PKB signaling nodes [16]. (ECS) extracellular space; the short red arrow (↑) indicates increased and the short black (↓) decreased experession of *A1*.

A key factor discriminating the intracellular concentration of Mg^2+^ ([Mg^2+^]) is the Na^+^/Mg^2+^ exchanger (NME) SLC41A1 (solute carrier family 41 member A1; further referred as to A1) [10,11]. Apart from occurring in the heart, it is abundantly expressed in the brain [12,13]. Its main function is to conduct Mg^2+^ efflux from the cell [9,10,11,13,14]. However, under Mg^2+^-influx-favoring conditions ([Mg^2+^]_extracellular_ >> [Mg^2+^]_intracellular_ and low [Na^+^]_extracellular_), it can also operate in reverse mode supporting Mg^2+^ influx [10,11,15]. The Mg^2+^ efflux capacity of the cell clearly depends on the quantity of functional A1 and also on the activity of the means regulating A1 performance [9,10,11,13,14]. The NME activity of A1 is modulated by cAMP-dependent PKA (activator) and indirectly by Akt/PKB (inhibitory effect on A1 via the activation of PDE3b and the consequent degradation of cAMP in the cell) [9,11,13]. Thus, any alterations leading to the increased efflux activity of A1 or to its increased abundance in the cytoplasmic membrane impose a direct risk for the excessive loss of Mg^2+^ from the cell resulting in intracellular Mg deficiency (Figure 1).

Both PKA and Akt/PKB have been implicated in the etiopathology of PD. Timmons and colleagues have identified a defective Akt/PKB signaling axis as a putative signaling pathway linked to the loss of dopaminergic neurons in PD [17]. Neuronal death (loss of dopaminergic neurons) in PD is also believed to be determined by the loss of balance between the antiapoptotic/prosurvival signaling of Akt/PKB and the proapoptotic/death-inducing signaling of JNK [18].

Interestingly, *A1* has been localized to the PD-associated locus *PARK16*, together with four other genes, namely *SLC45A3*, *NUCKS1*, *RAB7L1*, and *PM20D1* [19]. The rare A1 variant p.Ala350Val is putatively associated with PD [9,20]. It is insensitive to cAMP-PKA stimulation and it has significantly increased NME activity compared with wild type [9]. Thus, the p.Ala350Val variant of A1 is a gain-of-function mutant protein that possibly contributes to PD etiopathology by perpetuating excessive Mg^2+^ efflux from neurons, consequently resulting in the deterioration of neuronal energy metabolism and increased levels of oxidative stress [9,13,21]. Lin and colleagues have identified rare A1 variant p.Arg244His in a Taiwanese PD patient and have functionally characterized this amino acid substitution in vitro as leading to the loss of function of A1 [22].

SNP (single nucleotide polymorphism) rs11240569 (G > A; (-) strand NM_173854.5:c.339C>T; Thr [ACC] > Thr [ACT]) in the coding sequence of *A1* (leading to p.Thr113Thr) has been associated with PD [21]. The MATPFR (minor allele total population frequency range) for A here is as much as ≈30% (gnomAD browser beta, gnomad-old.broadinstitute.org/dbsnp/rs11240569; and ExAC, exac.broadinstitute.org/variant/1-205779231-G-A). Wang and coworkers have demonstrated, in a Chinese Han cohort from mainland China, that the G allele of the *A1* polymorphism rs11240569 reduces the risk of developing the idiopathic form of PD, and that probands with GG and AG genotypes have a reduced risk compared with those having the AA genotype [23]. A significant association between the rs11240569 polymorphism and the reduced risk of PD has also been found in an Iranian cohort, as demonstrated by the study of Madadi and colleagues [24].

Another PD-associated SNP in the coding sequence of *A1* is rs708727 (G>A; (-) strand NM_173854.5:c.756C>T; Asn [AAC] > Asn [AAT]; leading to p.Asn252Asn). The MATPFR for the A here is also as high as ≈30% (gnomAD browser beta, gnomad-old.broadinstitute.org/dbsnp/rs708727; and ExAC, exac.broadinstitute.org/variant/1-205767885-G-A). This SNP has been studied from the perspective of its possible association with PD by Tucci and colleagues [20]. They have shown that, in a United Kingdom cohort, no association between p.Asn252Asn and PD is present [20]. Interestingly, novel findings of Sachez-Mut and colleagues indicate that *PM20D1* (peptidase M20 domain containing 1) methylation and expression are dependent on the SNP rs708727, and that both the epigenetic regulation of *PM20D1* and the genetic variation at rs708727 are linked to Alzheimer’s disease [25].

A noncoding intron SNP of *A1*, rs823156 (A>G; NM_173854.5:c.), has been associated with PD by several genome-wide association studies (GWAS) [26,27,28]. The MATPFR for G here is as much as ≈23% (gnomAD browser beta, gnomad-old.broadinstitute.org/dbsnp/rs823156; and ExAC, http://exac.broadinstitute.org/variant/1-205764640-G-A). Chang and colleagues have shown by means of an additive model that, in the mainland China population, the minor allele at SNP rs823156 tends to reduce the risk of developing PD [28]. On the contrary, Yan and coworkers have been unable to confirm an association of the minor allele at SNP rs823156 with the reduced risk of developing PD in an Eastern China cohort [29]. A recent study conducted by Miyake and colleagues has led to the conclusion that, in the Japanese population, SNP rs823156 is significantly associated with PD [30]. Moreover, the authors have provided new evidence of an additive interaction between SNP rs823156 and smoking in relation to PD-susceptibility [30]. Chung and coworkers have associated SNP rs823156 with the susceptibility for PD also in the Korean population [31]. In a study conducted with north Spanish cohorts of PD patients and controls, Mata and colleagues have not confirmed the association between SNP rs823156 and susceptibility to PD seen prevalently in Asian populations [32]. The same is the case for the recent study of Gopalai and colleagues in the Malaysian population [33].

Slovaks belong to the West Slavic ethnic group [34]. In 2016, 412 PD patients per 100,000 inhabitants were under medical surveillance in Slovakia (National Health Information Center of Slovak Republic; www.nczisk.sk). In this study, we have examined the association of *A1* SNPs rs11240569, rs708727, and rs823156 with PD in Slovaks. Furthermore, we have examined PD diagnostic potential of the three *A1* SNPs in the Slovak population with the use of Random Forest (RF) machine-learning algorithm trained in four various modes.

## 2. Results

SNPs rs11240569, rs708727, and rs823156, all of which occur in gene *A1* located within the *PARK16* locus were included in the study. First, the allele and genotype count and frequency (fq) in PD and control cohorts were determined for each particular SNP (Table 1).

The total observed frequency range of the minor allele for each respective *A1* SNP in our study was either: (1) Comparable with MATPFR reported by gnomAD and ExAC databases, rs11240569 (G>A), fq_o_ (observed) vs. fq_r_ (reported) = 30.37% vs. 30%; or (2) higher than MATPFR reported by gnomAD and ExAC databases, rs708727 (G>A), 41.67% vs. 30%; or (3) lower than MATPFR reported by gnomAD and ExAC databases, rs823156 (A>G), 17.22% vs. 23%. No significant dissimilarities from Hardy-Weinberg equilibrium (HWE) were detected for each of the tested SNP in our PD and control cohorts (Table 2).

A summary of the odds ratios (OR) of minor alleles and of homozygotes and heterozygotes with two minor alleles of the three studied *A1* polymorphisms is presented in Table 3. In the case of the *A1* polymorphism rs11240569, the range of the confidence interval and X^2^ (*p*) values affirm that the A variant in the homozygous or heterozygous configuration is not associated with a risk of PD onset in our population, thus negating the association of rs11240569 with PD, such as that observed by Wang and colleagues in the Chinese population [23]. The same is the case for the *A1* polymorphism rs708727. Moreover, the assumed PD-protective role of the minor allele G at rs823156 has not been confirmed in our population (Table 3).

Next, we examined whether interactions among the three *A1* SNPs influenced susceptibility for developing PD in our population. Out of 27 possible combinations among the genotypes of the three SNPs, we have identified 11 as being present (Table 4). Only combination GG_(rs11240569)_/AG_(rs708727)_/AA_(rs823156)_ occurs significantly more frequently in the PD cohort (fq = 13%) than in the control cohort (fq = 5%) and is potentially associated with an increased risk of developing PD (X^2^ = 4.41, *p* = 0.04; Table 4) in the Slovak population. With respect to the GG_(rs11240569)_/AG_(rs708727)_/AA_(rs823156)_ combination, gender does not seem to play a role in PD or control cohorts as, among 20 carriers in the PD cohort, 9 were women (fq = 45%) and 11 were men (fq = 55%) and, among 6 controls, 3 were women (fq = 50%) and 3 were men (fq = 50%). We did not perform any statistical analysis because of the number of GG_(rs11240569)_/AG_(rs708727)_/AA_(rs823156)_ triplet-positive subjects in the groups of women and men in both PD and control cohorts. Among the remaining SNPs-associated genotype combinations, some triplets might be combinations potentially associated with PD; however, the numbers of subjects in the PD and control cohorts lay far below the statistical power of the analysis. The arcsine transformation parameters are presented in Table 4.

We also tested whether any genotypic combination in SNP duplets consisting of rs11240569 and rs708727, or rs11240569 and rs823156, or rs708727 and rs823156 was significantly more or less frequent in the PD cohort than in the control cohort. The data summarized in Table A2 demonstrate that frequency of none of the duplets is significantly different in the PD cohort. Similar to the analysis of the triplets, the low statistical power of the analysis should also be considered for the duplets.

**Figure 2:**
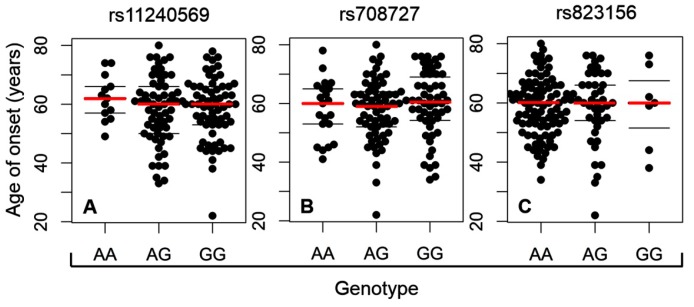
Correlation between age of onset of idiopathic PD and the particular genotypes at each tested *A1* SNP: (**A**) rs11240569, (**B**) rs708727, (**C**) rs823156. The correlation for each SNP involved 138 PD patients. Twelve patients out of 150 patients in PD cohort have been excluded from the correlation due to discrepancies about the age of PD onset in their documentation.

Age is considered as being among the most significant risk factors for the onset of the idiopathic form of PD [35]. Therefore, we were curious as to whether the age of onset of PD correlates with the presence of either the major or the minor homozygous genotype or the heterozygous genotype for each tested SNP. Figure 2A–C reveals that this is not the case for rs11240569, or rs708727, or rs823156; thus, for each of these SNPs, the age of onset has not correlated with a particular genotype (rs11240569, GG 58.4 ± 10.5 years, AG 58.0 ± 11.3, AA 62.1 ± 7.4 years; rs708727, GG 59.7 ± 11.1 years, AG 57.8 ± 10.4 years, AA 58.1 ± 9.8 years; and rs823156, GG 58.9 ± 12.9 years, AG 58.4 ± 12.3 years, AA 58.6 ± 9.6 years).

**Figure 3:**
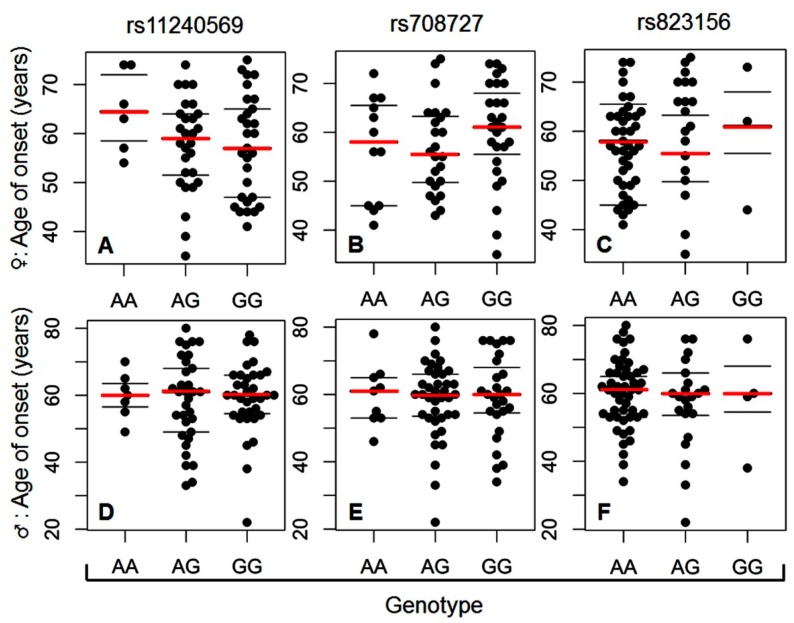
Correlation between age of onset of idiopathic PD and the particular genotypes at each tested *A1* SNP in the subgroup of female PD patients (**A–C**; (**A**) rs11240569, (**B**) rs708727, (**C**) rs823156; *N* = 63, 3 out of 66 patients in subcohort of PD females have been excluded from the correlation due to discrepancies about the age of PD onset in their documentation) and the subgroup of male PD patients (**D–F**; (**D**) rs11240569, (**E**) rs708727, (**F**) rs823156; *N* = 75, 9 out of 84 patients in subcohort of PD males have been excluded from the correlation due to discrepancies about the age of PD onset in their documentation).

Another factor attributable to the onset of idiopathic form of PD is gender, favoring men over women with ratio 1.5:1 [36]. Hence, we split the PD cohort into two groups based on gender (women versus men). Next, we correlated the age of PD onset with the presence of a particular genotype for each of the three tested SNPs in the same manner as described previously. The data presented in Figure 3 show that the age of onset of PD is not correlated with any particular genotype of any of the three SNPs in either the subcohort of PD women (A–C) or the subcohort of PD men (D–F).

**Figure 4:**
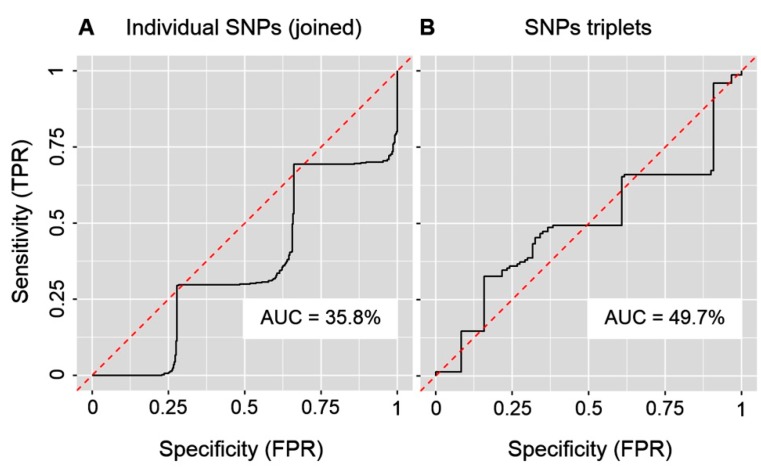
ROC (receiver operating characteristic) curves with the area under the ROC curve (AUC) for the machine-learning Random Forest algorithm with (**A**) the three individual *A1* SNPs (summarized in Table 1) as joined predictors and (**B**) with the 27 triplets of the three *A1* SNPs (summarized in Table 4) as predictors. Abbreviations: (TPR) true positive rate, (FPR) false positive rate.

**Figure A1:**
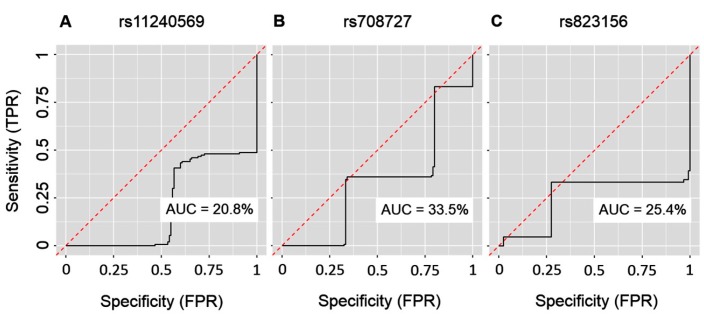
ROC (receiver operating characteristic) curves with the area under the ROC curve (AUC) for the machine-learning Random Forest algorithm with the three individual *A1* SNPs (summarized in Table 1) as isolated predictors: (**A**) rs11240569, (**B**) rs708727, (**C**) rs823156. Abbreviations: (TPR) true positive rate, (FPR) false positive rate.

**Figure A2:**
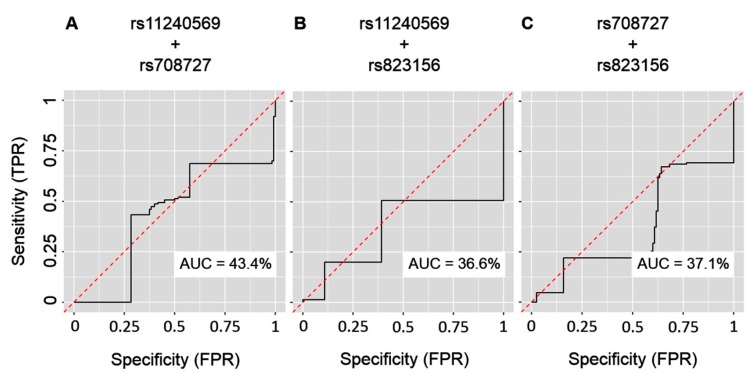
ROC (receiver operating characteristic) curves with the area under the ROC curve (AUC) for the machine-learning Random Forest algorithm with the nine genotypic duplets (summarized in Table A2) of the paired *A1* SNPs as predictors: (**A**) rs11240569 + rs708727, (**B**) rs11240569 + rs823156, (**C**) rs708727 + rs823156. Abbreviations: (TPR) true positive rate, (FPR) false positive rate.

In order to assess the ability of SNPs to discriminate between PD patients and controls, we have trained the RF machine-learning algorithm using our data [37,38,39]. The algorithm can evaluate the discriminative importance of individual SNPs by a technical construct known as graph depth. The predictive ability of the SNPs was visualized by ROC (receiver operating characteristic) curves and quantified by AUC (area under ROC curve). The perfect discriminative ability of predictors is associated with 100% AUC; 50% AUC (or less) corresponds to no discriminative ability. We have trained RF in four modes: (1) With each particular SNP (singlet; three genotypes: AA/AG/GG; Table 1) as predictor (three RF models, one for each SNP; data input per model N = 270 (150 PD patients + 120 controls)), (2) with the three SNPs (each SNP three genotypes: AA/AG/GG; Table 1) as joined predictors (one RF model; data input per model *N* = 810 (150 + 120) × 3), (3) with the nine genotypic duplets (Table A2) of the paired SNPs (rs11240569 + rs708727, or rs11240569 + rs823156, or rs708727 + rs823156) as predictors (three RF models, one for each pair of SNPs; data input per model *N* = 270 (150 + 120)), and (4) with the 27 genotypic triplets (Table 4) of the three SNPs as predictors (one RF model; data input per model *N* = 270 (150 + 120)). The first mode leads to an AUC of, 20.8% for rs11240569, 33.5% for rs708727 and 25.4% for rs823156 (Figure A1A–C), whereas the second gives an AUC of 35.8% (Figure 4A). The third mode gives an AUC of 43.4% for duplet rs11240569 + rs708727, 36.6% for duplet rs11240569 + rs823156 and 37.1% for duplet rs708727 + rs823156 (Figure A2A–C), and the fourth mode gives an AUC of 49.7% (Figure 4B). Hence, neither the evaluated *A1* SNPs in isolation, or together (used as joined predictors), or their duplets or triplets have the potential to serve as discriminators between healthy probands and PD patients and, with regard to PD, carry no predictive or diagnostic value in the Slovak population.

The findings from RF concerning diagnostic ability of the individual SNPs are in accord with the diagnostic ability of the histogram classifier, which leads the classification error of 49.6% for rs11240569, 44.8% for rs708727, and 49.3% for rs823156.

## 3. Discussion

Unsurprisingly, because of the importance of Mg^2+^ for mitochondrial homeostasis and energy production, its chronic cellular shortage has been considered among factors contributing to the onset of serious progressive metabolic and neurological diseases [5,7,8,9,14].

The involvement of Mg^2+^ deficiency in the onset and progression of PD is widely reported in the literature [40]. For example, Uitty and colleagues have shown that parkinsonian brains (PD and parkinsonism secondary to neurofibrillary tangle disease) have lower concentrations of Mg in the caudate nucleus and Cu (copper) in the *substantia nigra* than control brains [41]. The group of Montagna performed in vivo phosphorus magnetic resonance spectroscopy on the occipital lobes of 15 patients with multiple system atrophy (MSA; eight with olivopontocerebellar atrophy and seven with the striatonigral degeneration variant), 13 patients with idiopathic PD, and 16 age-matched healthy subjects [42]. In PD patients, they found a significantly increased content of P_i_, a decreased cytosolic Mg^2+^, and an unchanged concentration of phosphocreatine and pH. The discriminant power of 93% was determined for intracellular concentrations of Mg^2+^ and phosphocreatine between the MSA and PD groups [42]. Furthermore, Bocca and colleagues concluded in their study, which included 91 PD patients and 18 controls, that the progression and growing severity of PD were correlated with a decreasing concentration of Mg in the cerebrospinal fluid [43].

Until present, A1 is the only known and well-characterized cellular Mg^2+^-efflux system integral to the cytoplasmic membrane of the cell [13]. It fulfils all prerequisites of a carrier type of ion transporter and operates in the Na^+^/Mg^2+^ exchange mode [9,10,11]. The putatively PD-associated rare mutation causing the substitution of alanine with valine at position 350 of the A1 amino acid chain (p.Ala350Val) has been characterized as an A1 gain-of-function mutation leading to deregulated cellular Mg^2+^ wasting attributable to A1 breaking free of cAMP-PKA control [9]. Another rare A1 mutation, putatively associated with an early onset of PD in the Taiwanese population and leading to the substitution of arginine with histidine at position 244 of the A1 amino acid chain (p.Arg244His) has been shown to be a loss-of-function mutation, thus promoting the retention of Mg^2+^ in cells [22]. However, at the level of whole organism the loss of A1 function may largely impact on the ability of the body to absorb Mg^2+^ in gastro-intestinal tract and to re-absorb Mg^2+^ in kidneys. Thus, the gain-of-function but also loss-of-function A1 mutations may result in cellular/neuronal Mg^2+^ deficiency and potentially contribute to the onset and progression of PD [9,13,22].

PD-related *A1* SNP rs11240569 corresponds, at the protein level, to the synonymous substitution p.Thr113Thr [23]. Similarly, the case for PD-related *A1* SNP rs708727 corresponds to the synonymous substitution p.Asn252Asn [20]. Neither substitution changes the amino acid sequence of A1, and thus, they can be assumed not to affect qualitative and quantitative aspects of A1 performance in cells. Nevertheless, the minor allelic variants at rs11240569 and rs708727 might negatively influence the rates of A1 production and consequently the function of A1 in cells [44]. However, Mandt’s model of Mg^2+^-dependent endosomal A1 recycling through its N-terminal cytoplasmic domain accounting for an endosomally stored pool of A1 that is “always ready to perform” renders this option unlikely [45].

PD-associated SNP rs823156 is localized in the noncoding (intron) sequence of the *A1* gene [46]. Bai and coworkers have speculated that the function of A1 can be modified either by causing the alterative splicing of the gene or by altering the binding of transcription factors with *A1* [46]. Whereas these authors have considered alternative splicing as an unlikely option, they have shown, by *in silico* analysis, that A > G at rs823156 adds the putative transcription-factor-binding sites for NP-4 and RARA and eliminates the binding site for the transcription factor NFASC [45]. Influence of an A to G substitution in this SNP with regard to its possible bias in the processes of transcription and splicing of *A1* hnRNA or the hnRNA of any nearby or distant genes has not been experimentally studied as yet and thus, currently, cannot be excluded [47,48].

Wang and colleagues have associated the G allele of the *A1* polymorphism rs11240569 with the reduced risk of the onset of idiopathic PD in a Chinese Han cohort from mainland China and stated that probands with the GG and AG genotypes have a reduced risk compared with those with the AA genotype [23]. The protective role of the G allele has also been confirmed in the Iranian study conducted by Madadi and colleagues [24]. The investigation of Yan and coworkers conducted with Chinese PD patients (idiopathic form) of Han ethnicity has not confirmed the protective role of the G allele of the *A1* polymorphism rs11240569 found in the previous two studies [49]. Moreover, Tucci and colleagues have found no association between idiopathic PD and rs11240569 in their United Kingdom study [20]. Interestingly, the research groups of Wang and of Madadi have found the G allele to be minor in their PD/control cohorts with frequency ranges up to 35.9%/39.3% and 16%/20%, respectively, whereas Yan and colleagues have found the G allele to be major with a frequency range up to 58.05%/55.48% in their PD/control cohorts [23,24,49]. Tucci et al. have provided no data on G allele frequency but clearly indicate A allele at rs11240569 as being potentially PD-associated, thus assuming the G allele is major and the A allele minor in their study [20]. In our study, we have found the A allele to be minor at rs11240569 with a frequency range up to 30% in the PD cohort and 31% in the cohort of controls. The A allele frequency ranges fluctuate widely across various populations. According to the gnomAD database, the frequency range of A at rs11240569 in the East Asian population is up to 57.6%, in South Asian 37.15%, in European (non-Finnish) up to 29.21%, in European (Finnish) up to 28.34%, in Latino 19.47%, and in African 5.27%. Hence, differences in the frequency range of the allele A (which is, in some studies, minor and, in others, major) at rs11240569 in various races and ethnic groups might lie behind the contradicting outcomes regarding the association of this SNP with the idiopathic form of PD. Further association analyses of our *A1* SNP rs11240569 data clearly indicate that this *A1* polymorphism is not associated with an altered risk of developing the idiopathic form of PD in the Slovak population, and our conclusion regarding the association of rs11240569 with PD conforms with the conclusions of the studies of Yan et al. and Tucci et al. [20,49].

Another *A1* polymorphism rs708727 has not been established as being associated with PD by Tucci and colleagues in the United Kingdom association study [20]. Our data are in agreement with the conclusion of Tucci et al. and indicate that *A1* SNP rs708727 is not associated with a modified risk for onset of PD in Slovak population.

The minor allele G at *A1* SNP rs823156 has been associated with a reduced risk of PD onset in mainland Chinese (minor allele population frequency range (MAPFR) up to 16.1% in idiopathic PD and 20.6% in control cohorts), Japanese (MAPFR up to 14% in PD and 19% in control cohorts), and Korean (MAPFR up to 18% in PD and 21% in control cohorts) populations [28,30,31]. On the contrary, it is not associated with an altered risk for developing PD in eastern Chinese (MAPFR up to 18.58% in PD and 22.39% in control cohorts), Malaysian (MAPFR up to 19.31% in PD and 21.19% in control cohorts) and Spanish (MAPFR up to 16% in PD and 17% in control cohorts) populations [29,32,33]. The MAPFR for allele G at rs823156 in our PD (19%) and control (15%) cohorts clearly falls within the MAPFR range set for allele G in other studies. However, in our case, the minor allele at rs823156 seems interestingly to be more frequent in the PD cohort than in the control cohort, whereas in other previously reported studies, this was vice versa. Similar to two other tested SNPs, rs823156 in our study has not been established as being associated with PD, hence indicating a similar outcome as in the eastern Chinese, Malaysian, and Spanish studies [29,32,33].

Age and gender are two major factors known to contribute to the onset of idiopathic PD [35,36]. We have attempted to correlate age of PD onset with respective genotypes at particular tested SNPs. As depicted in Figure 2, no correlation has been found between the age of onset and any particular genotype at any of the three studied *A1* SNPs. The outcome of further stratification of PD patients into the subcohort of PD females and PD males and the correlation of age of onset of the disease with respective genotypes at particular tested SNPs in these subcohorts have revealed no positive outcomes (Figure 3). This leads us to assume that none of the three SNPs influences the age of onset of idiopathic PD in the cohort of our patients, irrespective of their gender.

Of the 27 possible triplet combinations among genotypes at rs11240569, rs708727, and rs823156, 11 were identified in both the PD and control cohorts. Only triplet GG_(rs11240569)_/AG_(rs708727)_/AA_(rs823156)_ was significantly (*p* = 0.04) more frequent among PD patients than in healthy controls and was projected as being associated with an increased risk of idiopathic PD onset in the Slovak population (OR = 2.92, 95 % CI: 1.14 to 7.53, *p* = 0.03, power of statistical significance = 65.3%). To our best knowledge, the association among the three *A1* SNPs tested here has not been performed in any other published study, thus rendering desirable the further testing of the association of the triplet GG_(rs11240569)_/AG_(rs708727)_/AA_(rs823156)_ with an increased risk for the onset of idiopathic PD in populations other than that of Slovakia.

Using the online sample size estimator (OSSE, http://osse.bii.a-star.edu.sg/calculation1.php), we have calculated that, for the *A1* SNP rs11240569 PD-association analysis, we would need around 33280 patients and the same number of control probands to achieve a power of 80% at the desired significance level of 5% and an estimated MAPFR of 30% in PD and 31% in control cohorts. To achieve the same power of the PD-association analysis at 5% significance for SNP rs708727 (MAPFR of 40% in PD and 43% in control cohorts), we would need 4234 patients and the same number of controls, and for SNP rs823156 (MAPFR of 19% in PD and 15% in control cohorts), we would need 1383 patients and the same number of controls. The numbers of study participants in our PD and control cohorts are clearly far below sample sizes sufficient to achieve the desirable statistical power. Since large samples, even when available, are associated with the *p*-value problem [50], we have further utilized a machine-learning approach [51,52], which demands lower sample sizes (cf. Hoeffding’s inequality, Vapnik-Chervonenkis inequality [52,53]) to evaluate the discriminative importance of individual SNPs or their duplets, or triplets.

Statistical analysis permits inferences to be drawn from a sample to the level of the population from which the sample was drawn. In the case of our study, we can infer that the triplet of SNPs with the genotypic combination GG_(rs11240569)_/AG_(rs708727)_/AA_(rs823156)_ is statistically significantly more frequent in the population of Slovak PD patients than in controls (Table 4) and might be potentially associated with an increased risk of developing PD. Machine-learning offers a different perspective of a data set than does statistical inference [52]. Indeed, machine-learning permits the discriminative ability of SNPs between PD patients and controls to be quantified. In this respect, the three studied SNPs, either in isolation or together, or as a duplets or triplets, have failed to be of any interest, as their discriminative ability is essentially zero (Figure 4, Figure A1 and Figure A2), since the AUC are well below or at the best close to 50%.

## 4. Materials and Methods

### 4.1. Study Participants

A total of 270 people were recruited for the study (150 PD cases and 120 control cases). All PD cases were diagnosed by neurologists at the Neurology Clinic of University Hospital in Martin (UNM, Martin, Slovakia) with the idiopathic form of PD. Diagnostics followed the MDS (Movement Disorders Society) clinical diagnostic criteria for PD [54]. Cases with a positive family history of PD were excluded from the study. Participating PD patients were in stages 1 through 4 of the Hoehn and Yahr scale [55]. All PD patients during the time of material sampling were on the standard PD treatment involving levodopa, dopamine agonists, catechol-O-methyl transferase (COMT) inhibitors, monoamine oxidase B (MAO-B) inhibitors, amantadine, anticholinergic medication, and DBS (deep brain stimulation). The average age of PD onset was 58.6 ± 10.6 years of age (if not stated otherwise and where applicable, the data throughout the text are presented as value ± standard deviation (SD)), with the youngest case being diagnosed at 22 years of age and the oldest case at 80 years of age. The average duration of the disease was 8.44 ± 5.73 years. PD is up to 1.5 times more frequent in men than women [36]. The female to male (F:M) ratio in our PD cohort was 1:1.28. The control cohort consisted of outward and inward patients from the Clinic of Occupational Medicine and Toxicology (UNM, Martin, Slovakia). The F:M ratio in the control group was 1:1.45. The average age of control probands was 59.2 ± 4.68 years. Neurodegenerative, neuropsychiatric diseases, *diabetes mellitus*, and osteoporosis (all diseases putatively associated with disturbed A1 expression and A1 regulation) served as exclusion criteria for the control group probands. No studies examining an association of SNPs rs11240569, rs708727, and rs823156 with these diseases are available in peer-reviewed literature. The study was approved by the Ethics Committee (EC) of Jessenius Faculty of Medicine. The approval was recorded under ID: EK 124/2018 on 13 December2018 by the EC board. All participants signed written informed consent forms.

### 4.2. Sample Processing

Blood of PD patients and control probands was collected in sterile EDTA-treated sampling tubes by the staff of UNM. Blood samples were subsequently centrifuged at 450× *g* and 4 °C for 20 min. After plasma aspiration, genomic DNA was isolated from cellular content by using the Wizard^®^ Genomic DNA Purification Kit (Promega Corporation, Maddison, WI, USA) according to the manufacturer’s protocol. Isolated DNA was stored at −45 °C until analysis.

### 4.3. Genotyping

SNPs rs11240569, rs823156, and rs708727 were selected for this study. Genotyping was performed with TaqMan^®^ genotyping probes C_34251_20/rs11240569, C_375742_10/rs823156, and C_9238453_10/rs708727 (all Thermo Fisher Scientific, Waltham, MA, USA). The TaqMan qPCR genotyping reaction was performed according to the manufacturer´s protocol. Briefly, 10 ng genomic DNA was mixed with TaqMan probe, HOT FIREPol^®^ Probe qPCR Mix Plus without ROX (Solis BioDyne, Teaduspargi, Estonia), and PCR grade water. Samples were analyzed in duplicates on 96-well plate. All samples underwent 40 PCR cycles. Samples were genotyped with a Viia7 Real Time PCR cycler (Applied Biosystems, Foster City, CA, USA). Genotypes were determined with the TaqMan Genotyping tool of Viia 7 Software (Applied Biosystems, Foster City, CA, USA). Data were subsequently exported for further statistical analysis.

### 4.4. Biostatistical Analysis

The hypothesis of the Hardy-Weinberg equilibrium was tested with the OEGE HWE calculator including an analysis for ascertainment bias [56]. Odds ratios were computed by using R ver. 3.5.2 (R Core Team (2018). R: A language and environment for statistical computing. R Foundation for Statistical Computing, Vienna, Austria, https://www.R-project.org), and MEDCALC^®^ freeware (https://www.medcalc.org/calc/odds_ratio.php) and, where applicable, cross-checked with AssociatORRR CC software by JBG Hayesmoore (https://www.genecalculators.net/associatorrr-cc.html) and OEGE OR calculator software (http://www.oege.org/software/orcalc.html). The hypothesis of equality of population proportions of a joint genotype in PD patients and in controls was tested by the two-sample test of equality of proportions with continuity correction. Power analysis for the two-sample test of equality of proportions was performed by using R library pwr assuming a power of 0.8 (Stephane Champely et al. (2018). pwr: Basic Functions for Power Analysis. R package version 1.2-2., https://CRAN.R-project.org/package=pwr). For each SNP, the hypothesis of the equality of population mean age of onset among the three genotypes was tested by the ANOVA test. Several predictive models of the case-control status were built using the RF algorithm, using different sets of predictors: (1) Individual SNP as predictor (three RF models), (2) all the three SNPs as predictors (single RF model), (3) duplets of SNPs as predictors (three different duplets of SNPs, hence, three RF models), (4) triplets of SNPs as predictors (single RF model). The first model was used to explore the predictive power of the individual SNPs; the second one was used to leverage the interaction between SNPs; the third and fourth models explored the predictive power of duplets, triplets; respectively. The discriminative ability of a model was assessed by ROC curves and quantified by the AUC. Machine-learning was performed in R, with the aid of the libraries: (1) Beeswarm (Aron Eklund (2016). beeswarm: The Bee Swarm Plot, an Alternative to Stripchart. R package version 0.2.3., https://CRAN.R-project.org/package=beeswarm), (2) randomForestSRC (Ishwaran Hemant (2019). Random Forests for Survival, Regression, and Classification (RF-SRC)), (3) R package version 2.8.0., (https://cran.r-project.org/web/packages/randomForestSRC/randomForestSRC.pdf), (4) ggRandomForests (John Ehrlinger (2016). ggRandomForests: Visually Exploring Random Forests. R package version 2.0.1., https://CRAN.R-project.org/package=ggRandomForests), and (5) pwr.

## 5. Conclusions

In summary, our data suggest no association of *A1* SNPs rs11240569, rs708727, and rs823156, when considered in isolation, with idiopathic PD in the Slovak population. However, the triplet of SNPs with the genotypic combination GG_(rs11240569)_/AG_(rs708727)_/AA_(rs823156)_ might indeed be associated with an increased risk of spontaneous PD onset among Slovaks. From a statistical point of view, our data have to be interpreted cautiously because of the small sample size in both the PD and control cohorts and the low statistical power of the performed analyses. Nevertheless, the machine-learning approach, which demands considerably smaller sample sizes than conventional frequentist statistics or approximate Bayesian computation [57,58], has allowed us to examine the ability of particular *A1* SNPs or their duplets and triplets in various genotypic combinations to discriminate between PD patients and controls. In all instances, the machine-learning approach leads to essentially a zero result, indicating no diagnostic relevance (in terms of PD diagnostics and prediction) of SNPs rs11240569, rs708727, and rs823156 in the Slovak population. From the larger perspective, our data further substantiate the proposed ethnic and interracial differences between diverse populations with regard to the statistical association of the studied *A1* SNPs with the idiopathic form of PD [29,32,33,49]. Furthermore, the broader utilization of machine-learning in the assessment of the clinical relevance of various SNPs should be encouraged because, despite the statistical significance of the association of any SNP with a disease condition, which is entirely dependent on the sample size, its diagnostic power (to discriminate ill from healthy individual) might still be negligible.

## Figures and Tables

**Table 1:** Allele and genotype count and frequency in PD and control cohorts for tested *A1* SNPs.

SNP	Cohort	Alelle	Count	fq(%)	Genotype	Count	fq(%)
rs11240569	PD	G	210	70	GG	73	48
		A	90	30	AG	64	43
					AA	13	9
	C	G	166	69	GG	57	48
		A	74	31	AG	52	43
					AA	11	9
rs708727	PD	G	179	60	GG	54	36
		A	121	40	AG	71	47
					AA	25	17
	C	G	136	57	GG	40	33
		A	104	43	AG	56	47
					AA	24	20
rs823156	PD	A	243	81	AA	100	67
		G	57	19	AG	43	29
					GG	7	5
	C	A	204	85	AA	87	73
		G	36	15	AG	30	25
					GG	3	2

(C) control, (fq) frequency, (PD) Parkinson’s disease, (SNP) single nucleotide polymorphism.

**Table 2:** Genotype distribution of all tested *A1* SNPs in PD and control cohorts conforms to Hardy-Weinberg equilibrium.

SNP	rs11240569 (G > A) Cohort		rs708727 (G > A) Cohort		rs823156 (A > G) Cohort	
	PD N (O/E)	C N (O/E)	PD N (O/E)	C N (O/E)	PD N (O/E)	C N (O/E)
GG (com.)	73/73.5	57/57.4				
AG	64/63	52/51.2				
AA (rar.)	13/13.5	11/11.4				
*X^2^*	0.04	0.03				
*p*-val	0.85	0.86				
GG (com.)			54/53.4	40/38.53		
AG			71/72.2	56/58.93		
AA (rar.)			25/24.4	24/22.54		
*X^2^*			0.04	0.30		
*p*-val			0.84	0.59		
AA (com.)					100/98.42	87/86.7
AG					43/46.16	30/30.6
GG (rar.)					7/5.42	3/2.7
*X^2^*					0.71	0.05
*p*-val					0.40	0.83

(com.) common, (C) control, (E) expected, (N) number of individuals, (O) observed, (*p*-val) *p* value, (PD) Parkinson’s disease, (rar.) rare, (SNP) single nucleotide polymorphism.

**Table 3:** Odds ratios of minor alleles and genotypes containing minor allele at particular *A1* SNPs.

			95% CI					95% CI		
SNP	MA	OR	ll	ul	*p*-val	genotype	OR	ll	uL	*p*-val
rs11240569 (G > A)	A	1.04	0.71	1.53	0.85	AA	1.08	0.41	2.84	1.00
						AG	1.04	0.61	1.78	0.90
rs708727 (G > A)	A	1.13	0.79	1.62	0.48	AA	1.29	0.61	2.75	0.48
						AG	1.06	0.60	1.89	0.89
rs823156 (A > G)	G	1.33	0.82	2.17	0.25	GG	2.02	0.45	12.5	0.35
						AG	1.62	0.34	10.5	0.73

(CI) confidence interval, (ll) lower limit, (MA) minor allele, (OR) odds ratio, (*p*-val) *p* value, (SNP) single nucleotide polymorphism, (ul) upper limit.

**Table 4:** The equality of population proportions of a joint genotype in PD patients and in controls: various genotypic combinations in the triplets of *A1* SNPs.

PD Cohort				C Cohort							Arcsine Transformation *			
I	II	III	N	I	II	III	N	*X^2^*	df	*p*-val	d	N	sl	pw
AA	AA	AA	0	AA	AA	AA	0							
AG	AA	AA	0	AG	AA	AA	0							
GG	AA	AA	25	GG	AA	AA	24	0.30	1	0.58	0.09	1056	0.05	0.8
AA	AG	AA	0	AA	AG	AA	0							
AG	AG	AA	34	AG	AG	AA	36	1.51	1	0.22	0.17	282	0.05	0.8
GG	AG	AA	20	GG	AG	AA	6	4.41	1	0.04	0.30	89	0.05	0.8
AA	GG	AA	11	AA	GG	AA	11	0.11	1	0.75	0.07	1762	0.05	0.8
AG	GG	AA	9	AG	GG	AA	6	0.01	1	0.93	0.04	4071	0.05	0.8
GG	GG	AA	1	GG	GG	AA	4	1.35	1	0.25	0.20	189	0.05	0.8
AA	AA	AG	0	AA	AA	AG	0							
AG	AA	AG	0	AG	AA	AG	0							
GG	AA	AG	0	GG	AA	AG	0							
AA	AG	AG	0	AA	AG	AG	0							
AG	AG	AG	1	AG	AG	AG	1	0.00	1	1	0.02	20968	0.05	0.8
GG	AG	AG	16	GG	AG	AG	13	0.00	1	1	0.01	271094	0.05	0.8
AA	GG	AG	2	AA	GG	AG	0							
AG	GG	AG	20	AG	GG	AG	9	1.80	1	0.18	0.19	211	0.05	0.8
GG	GG	AG	4	GG	GG	AG	7	1.00	1	0.32	0.16	307	0.05	0.8
AA	AA	GG	0	AA	AA	GG	0							
AG	AA	GG	0	AG	AA	GG	0							
GG	AA	GG	0	GG	AA	GG	0							
AA	AG	GG	0	AA	AG	GG	0							
AG	AG	GG	0	AG	AG	GG	0							
GG	AG	GG	0	GG	AG	GG	0							
AA	GG	GG	0	AA	GG	GG	0							
AG	GG	GG	0	AG	GG	GG	0							
GG	GG	GG	7	GG	GG	GG	3	0.38	1	0.54	0.12	564	0.05	0.8

* proportion power calculation for binomial distribution. (I) rs11240569, (II) rs708727, (III) rs823156, (d) Cohen’s d, (C) control, (df) degrees of freedom, (N) number of individuals, (*p*-val) *p* value, (PD) Parkinson’s disease, (pw) power of test, (sl) significance level, (SNP) single nucleotide polymorphism.

## Abbreviations

A1	SLC41A1
Akt/PKB	Akt/Protein kinase B
AUC	Area under curve
cAMP	3’,5’-cyclic adenosine monophosphate
CI	Confidence interval
DBS	Deep brain stimulation
EGF	Epidermal growth factor
fq	Frequency
HWE	Hardy-Weinberg equilibrium
JNK	c-Jun N-terminal kinase
MAPFR	Minor allele population frequency range
MATPFR	Minor allele total population frequency range
ML	Machine-learning
MSA	Multiple system atrophy
NME	Na^+^/Mg^2+^ exchanger
NUCKS1	Nuclear ubiquitous casein and cyclin-dependent kinases substrate
OR	Odds ratio
OSSE	Online sample size estimator
*PARK19*	PD-associated locus 16
PD	Parkinson’s disease
Pde3b	Phosphodiesterase 3b
PDGF	Platelet-derived growth factor
PI3K	Phosphoinositide 3-kinase (phosphatidylinositol 3-kinase)
PKA	Protein kinase A
PM20D1	Peptidase M20 domain containing 1
*p*-val	*p* value
RAB7L1	Ras-related protein Rab-7L1
RF	Random Forest
ROC	Receiver operating characteristic
RTK	Receptor tyrosine kinases
SLC41A1	Solute carrier family 41, member A1
SLC45A3	Solute carrier family 45, member A3 (prostate cancer-associated protein 6/prostein)
SNP	Single nucleotide polymorphism

## Appendix A

**Table A1:** Overview of symptoms/complications associated with idiopathic PD *.

**Motor Complications of PD**	
**Early**	**Late**
Difficult turning in bed	Motor fluctuations
Frozen shoulder Stiffness, numbness or pain in limb Micrographia	Choreiformic dyskinesia Gait freezing Falls
Difficulty with fine finger movements Tremor of hand, jaw, foot Decreased facial expression Decreased arm swing, dragging a leg Soft voice	
**Non-motor complications of PD**	
**Early**	**Late**
Constipation	Dysphagia
REM sleep behaviour disorder	Neuropsychiatric symptoms
Depression	Autonomic disturbances
Olfaction impairment	Seborrheic dermatitis

* The table is adopted from Rizek et al. [59].

**Table A2:** Equality of population proportions of a joint genotype in PD patients and in controls: various genotypic combinations in the duplets of *A1* SNPs.

PD Cohort			C Cohort						Arcsine Transformation *			
**I**	**II**	**N**	**I**	**II**	**N**	***X^2^***	**df**	***p*-val**	**d**	**N**	**sl**	**pw**
AA	AA	0	AA	AA	0							
AA	AG	0	AA	AG	0							
AA	GG	13	AA	GG	11	0.00^#^	1	1.00	0.02	25493	0.5	0.8
AG	AA	0	AG	AA	0							
AG	AG	35	AG	AG	37	1.55	1	0.21	0.17	274	0.5	0.8
AG	GG	29	AG	GG	15	1.81	1	0.18	0.19	223	0.5	0.8
GG	AA	25	GG	AA	24	0.30	1	0.58	0.09	1056	0.5	0.8
GG	AG	36	GG	AG	19	2.26	1	0.13	0.21	186	0.5	0.8
GG	GG	12	GG	GG	14	0.65	1	0.42	0.12	513	0.5	0.8
**I**	**III**		**I**	**III**								
AA	AA	11	AA	AA	11	0.11	1	0.75	0.07	1762	0.5	0.8
AA	AG	2	AA	AG	0							
AA	GG	0	AA	GG	0							
AG	AA	43	AG	AA	42	0.96	1	0.33	0.14	424	0.5	0.8
AG	AG	21	AG	AG	10	1.59	1	0.21	0.18	239	0.5	0.8
AG	GG	0	AG	GG	0							
GG	AA	46	GG	AA	34	0.08	1	0.78	0.05	2997	0.5	0.8
GG	AG	20	GG	AG	20	0.35	1	0.55	0.09	898	0.5	0.8
GG	GG	7	GG	GG	3	0.38	1	0.54	0.12	564	0.5	0.8
**II**	**III**		**II**	**III**								
AA	AA	25	AA	AA	24	0.30	1	0.58	0.09	1055	0.5	0.8
AA	AG	0	AA	AG	0							
AA	GG	0	AA	GG	0							
AG	AA	54	AG	AA	42	2 × 10^−3^	1	0.97	0.02	17971	0.5	0.8
AG	AG	17	AG	AG	14	0.00 ^##^	1	1.00	0.01	71890	0.5	0.8
AG	GG	0	AG	GG	0							
GG	AA	21	GG	AA	21	0.38	1	0.54	0.10	847	0.5	0.8
GG	AG	26	GG	AG	16	0.54	1	0.46	0.11	634	0.5	0.8
GG	GG	7	GG	GG	3	0.38	1	0.54	0.12	564	0.5	0.8

* proportion power calculation for binomial distribution ^#^ 1.04 × 10^−30 ##^ 7.93 × 10^−31^. (I) rs11240569, (II) rs708727, (III) rs823156, (C) control, (d) Cohen’s d, (df) degrees of freedom, (N) number of individuals, (*p*-val) *p* value, (PD) Parkinson’s disease, (pw) power of test, (sl) significance level, (SNP) single nucleotide polymorphism.
